# Case Report: Laparoscopic Approach for Orchiopexy in a 26-Year-Old Man with Accidentally Discovered Persistent Müllerian Duct Syndrome and Bilateral Undescended Testis

**DOI:** 10.1089/cren.2018.0035

**Published:** 2018-05-01

**Authors:** Mohamed Essam Noureldin, Ahmed Mohamed Tawfeek, Hassan S. Shaker

**Affiliations:** ^1^ Department of Urology, James Paget University Hospital, Norwich, United Kingdom.; ^2^ Department of Urology, Ain Shams University Hospitals, Cairo, Egypt.

**Keywords:** laparoscopic orchiopexy, persistent Müllerian duct syndrome, undescended testis in adult

## Abstract

***Background:*** Persistent Müllerian duct syndrome (PMDS) is not a common form of disorder of sex development in which Müllerian duct derivatives (fallopian tubes, uterus, and the proximal vagina) are present in an otherwise normally differentiated 46 XY male. In most of cases, the challenge comes in the procedure of orchiopexy.

***Case Presentation:*** We report a case of a 26-year-old man with PMDS. It was accidentally discovered when the patient presented to our outpatient clinic concerning about his empty scrotum as a premarital check. Diagnostic laparoscopy discovered Mullerian remnants in the form of uterus, cervix, and fallopian tubes with two attached testes to the fallopian tubes. Staged laparoscopic orchiopexy was done. We discuss the presentation, the management of this case in the literature, and our intervention.

***Conclusion:*** PMDS is not a common condition. Several concerns present in the management of these cases. Malignant transformation of the testis is the main risk facing those patients. Few literature studies discussed the risk of changing of these remnants into malignant tissue. Thus discussion with the patient, tissue histopathology, expert opinions, and literature review are the main clues in management of such cases.

## Introduction

Persistent Müllerian duct syndrome (PMDS) is a condition that describes patients having remnants of the Müllerian duct such as the uterus, cervix, and fallopian tubes although being XY karyotype. In addition, it is usually associated with bilateral undescended testes or more seriously testicular tumors of these abdominal testes as the first presentation. It is so uncommon condition as only 260 cases are published in the literature.^1^ A case of PMDS in an adult male with bilateral undescended testis was encountered by our team and is discussed in this report.

## Case presentation

### History

A 26-year-old male patient was referred to our outpatient clinic in Ain Shams University Hospitals concerning about his empty scrotum since birth. The patient had no erectile problems. This was also not associated with any urologic symptoms. There was no family history of relevant condition. There was no other medical or surgical history of relevant importance.

### Examination

No abnormalities were detected except for his hypoplastic scrotum. Otherwise, he had an average build with good secondary male sexual characters. He had normal penile length as regard the age. Urethral meatus showed no abnormalities as regard the site. Neither of the testes could be felt in the scrotum or in the inguinal canal. Laboratory work-up showed mildly elevated FSH 14 mIU/mL (normal range = 1.5 to 12.4 mIU/mL), LH 12 nmol/L (normal range = 10.5 to 35 nmol/L), testosterone 400 ng/dL (normal range = 270 to 1070 ng/dL), and prolactin 4 ng/dL (normal range <20 ng/dL). Abdominopelvic ultrasonography detected bilateral abdominal testes in the iliac region. Karyotyping was 46 XY.

### Intervention

**FIG. 1.**
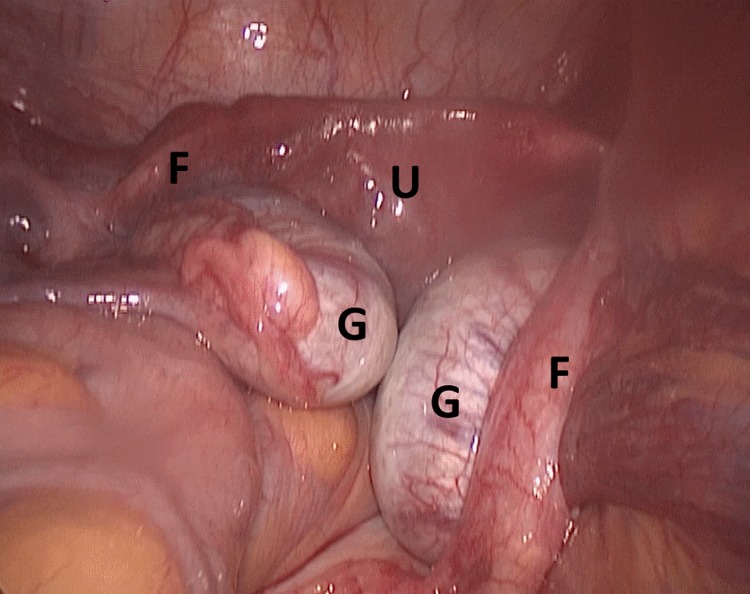
Diagnostic laparoscopy showed Müllerian remnants in the form of uterus (U), cervix, two fallopian tubes (F), and two gonads (G).

Diagnostic laparoscopy revealed midline abnormal structures in the form of uterus and cervix with two gonads attached to fallopian tube on each side (Fig. 1). Consequently, gonadal biopsies were taken that showed Sertoli cells without any spermatogenetic cells. It also showed Leydig cells hyperplasia.

The condition was discussed with the patient. He consented to orchiectomy and excision of all the Müllerian remnants or orchiopexy for early detection of the testicular tumors and keeping parts of the remnants with unsupported evidence for malignant transformation of these remnants. Accordingly, he opted for orchiopexy. The abdominal position of the testes required staged Stephen Fowler operation for the orchiopexy as the gonadal vessels were tethering the testes in their position.

**FIG. 2.**
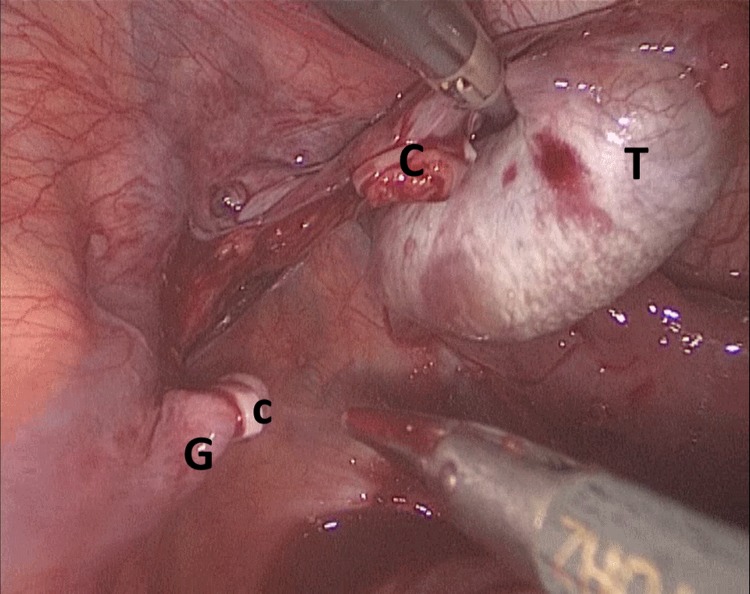
First stage Fowler Stephen procedure, in which clipping (C) of the gonadal (G) vessel was done allowing the testis (T) to receive blood supply from the artery of the vas only.

In the first stage, with two 10 mm ports and one 5 mm laparoscopic ports, any dissection around the testis was avoided. Clipping of the gonadal vessels was done using two Hem-o-lok polymer ligating clips in each side and testes were left in the abdominal position (Fig. 2).

Three months later, pelvic duplex ultrasonography was formed before the procedure, which confirmed the adequate vascularity of the testes. The same abdominal access was used as in the first stage. Blunt and cold knife lateral dissection of the testis and the fallopian tubes was done without any distal dissection of the fallopian tube. Dissection of the vas from the fallopian tubes was avoided.

Adequate length was gained by this dissection without any manipulation or incision in the uterus.

**FIG. 3.**
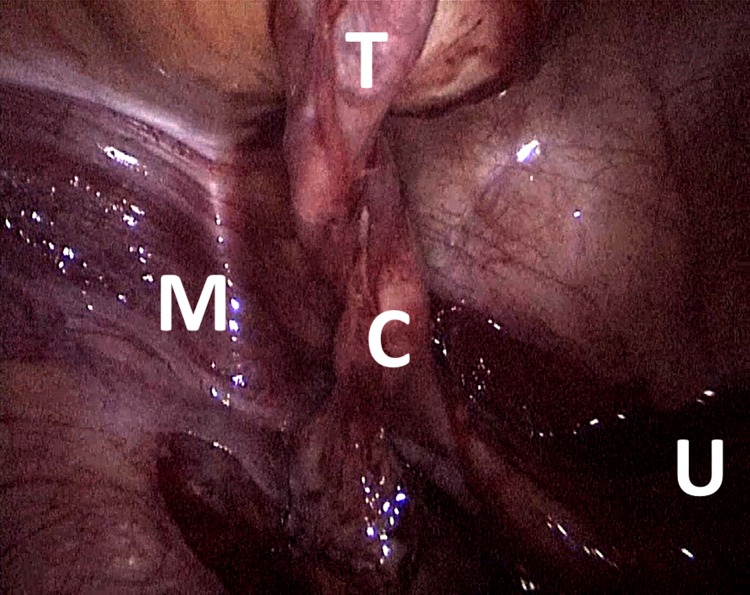
Prentiss's maneuver, the uterus (U) appeared in the midline without splitting with the cord and fallopian tube (C) getting out lateral to the medial umbilical ligament (M) with the testis (T) attached.

Additional length was obtained with the Prentiss maneuver, in which the testis was passed behind the inferior epigastric vessels, allowing for a more medial position of the cord. A 2-cm external incision in the scrotum was done and another incision in the peritoneum between the bladder and the medial umbilical ligament just lateral to the median umbilical ligament was done. The testis was grasped and replaced in the scrotum without any tension on the cord (Fig. 3). The procedure took about 50 minutes and required no blood transfusion.

### Follow-up and outcomes

The patient was discharged 24 hours later and scrotal duplex 1, 2, and 4 weeks later showed adequate blood supply for both testes in the scrotum. The patient was scheduled for annual monitoring of the testes in the scrotum and the remnants of the Müllerian duct for any malignant transformation

## Discussion

Embryologically, the disappearance of the Müllerian ducts in male in the 7th week of gestational age is closely related to the testicular descent. Thus, the absence of the anti-Müllerian hormone or resistance of one of the receptors will prevent the downward migration of the testis. However, in some cases the testis could be found in other places rather than its abdominal position and that is why Clarnette and coworkers in 1997 divided PMDS into three categories according to testicular position: the majority (60% to 70%) with bilateral intra-abdominal testes in a position analogous to ovaries(female type), a smaller group (20% to 30%) in which one testis is found in a hernial sac, and the smallest group (10%) in which both testes are located in the same hernia sac along with the fallopian tubes and uterus.^1^

Consequently, the main challenge that arises in these cases is the orchiopexy procedure owing to the presence of the usual oncologic risk associated with cryptorchidism in this syndrome in addition to the reported cases that had Müllerian remnant tumors. Another concern arising during orchiopexy is the testicular viability through securing adequate blood supply to the testis and preserving the vasa deferentia. Nevertheless, this is not feasible in the PMDS as the vasa deferentia are closely adherent to the uterus and proximal vagina in these cases and the testes are usually tethered by the Müllerian remnants, thus dissecting the vasa from the fallopian tubes may damage it and affect the fertility. By the same concept, when orchiopexy is done only for early detection of testicular tumors in older males, those cases may also require meticulous preservation of the vas especially after the Stephen Fowler procedure as the testis will depend on the artery of the vas for its blood supply. Therefore, preserving the vas is of importance in PMDS cases either for infertility or for vascularity purposes.

Consequently, some authors advocate that midline splitting of the Müllerian remnants and mucosal excision will give more length and allow feasible orchiopexy. Excision of the remnants and effective orchiopexy with delicate dissection of the vasa was reported by few authors. However, testicular atrophy was reported in some of those cases.^2^

According to the limited amount of literature, there is not a standard practice for orchiopexy in PMDS. It could be managed by a single-stage procedure or a two-stage procedure including gonadal biopsies in first stage followed by Müllerian remnants excision or dissection and orchidopexy in the second stage. In contrast, the persistent Müllerian structures can be divided minimally to allow placement of the testis in the scrotum or incised in the midline or even excised totally. At this point, a staged procedure could be considered if manipulation of the Müllerian structures puts the vas in jeopardy, so Fowler Stephens procedure or microvascular auto transplantation will be helpful.^3^

## Conclusion

PMDS is not a common condition and most urologists will not encounter it during their career. However, a few points could be learned from our case. The release of the fallopian tubes added marvelously to the length of the cord, and midline splitting of the uterus was not required although it was planned based on reviewing the literature. Gonadal biopsies, intraoperative photographs, senior staff consultation, and patient counseling were the corner stones in effective management of this condition.

## Abbreviation Used

PMDS: persistent Müllerian duct syndrome
